# Radioactive Iodine Remnant Ablation: The Beta-knife Completion Thyroidectomy

**DOI:** 10.4274/2017.26.suppl.03

**Published:** 2017-01-09

**Authors:** Seza Gulec, Russ Kuker

**Affiliations:** 1 Florida International University Herbert Wertheim College of Medicine, Departments of Surgery and Nuclear Medicine, Miami, USA; 2 University of Miami Miller School of Medicine, Department of Radiology, Miami, USA

**Keywords:** radioactive iodine, ablation, thyroid cancer, thyroid remnant, I-131, I-124, dosimetry, beta knife

## Abstract

The rationale and objectives for radioactive iodine (RAI) ablation remain perplexing to many. This review addresses the meaning, clinical context and the goals of “ablation”: the RAI treatment after a total thyroidectomy. This article also aims to clarify the definition of a total thyroidectomy and how a thyroid remnant can introduce a confounding factor in the postoperative management of patients with differentiated thyroid cancer. The implications of an existing thyroid remnant on RAI diagnostic imaging and serum thyroglobulin levels are discussed. This review provides a rational approach validating the utility of RAI remnant ablation regardless of the patient’s risk stratification.

## Abstract (tr)

Radyoaktif iyot (RAİ) ablasyon kavramı mantığının ve hedeflerinin birçok kişi için hala yeterince açık olmadığını görüyoruz. Bu makalede total tiroidektomiyi takiben yapılan RAİ “ablasyon” girişiminin anlamı, hedefleri ve klinik değeri izah edilmektedir. Gerçekçi anlamda total tiroidektominin RAİ ablasyonu ile mümkün olduğu tezini savunuyor ve destekleyen verileri sistematik olarak sunuyoruz.

## INTRODUCTION

The role for radioactive iodine (RAI) in the management of metastatic differentiated thyroid cancer (DTC) is indisputable. Its utilization postoperatively as part of initial treatment, however, is still a matter of debate. The 2015 American Thyroid Association (ATA) guidelines is a 133-page document written in a dissertation format (1). The rationale for each recommendation is discussed, in detail, in a scholarly fashion, but yet, there is a sense of ambiguity in defining the indications for administration of RAI ablation. This review will discuss the ATA recommendations from a perspective that accounts surgical considerations, nuclear medicine principles, and genomic developments.

## THE ROLE OF RAI (INCLUDING REMNANT ABLATION, ADJUVANT THERAPY, OR THERAPY FOR PERSISTENT DISEASE) AFTER THYROIDECTOMY IN THE PRIMARY MANAGEMENT OF DIFFERENTIATED THYROID CANCER

**2015 ATA Recommendation 51**

A) RAI remnant ablation is not routinely recommended after thyroidectomy for ATA low-risk DTC patients. Consideration of specific features of the individual patient that could modulate recurrence risk, disease follow-up implications, and patient preferences are relevant to RAI decision-making (Weak recommendation, low-quality evidence).

B) RAI remnant ablation is not routinely recommended after lobectomy or total thyroidectomy for patients with unifocal papillary microcarcinoma, in the absence of other adverse features (Strong recommendation, moderate-quality evidence).

C) RAI remnant ablation is not routinely recommended after thyroidectomy for patients with multifocal papillary microcarcinoma in the absence of other adverse features. Consideration of specific features of the individual patient that could modulate recurrence risk, disease follow-up implications, and patient preferences are relevant to RAI decision-making (Weak recommendation, low-quality evidence).

D) RAI adjuvant therapy should be considered after total thyroidectomy in ATA intermediate-risk level DTC patients (Weak recommendation, low-quality evidence).

E) RAI adjuvant therapy is routinely recommended after total thyroidectomy for ATA high-risk DTC patients (Strong recommendation, moderate-quality evidence).

RAI treatment is given in three distinct settings with different clinical indications (intents);

1) Ablation of the remnant

2) Adjuvant treatment for residual disease or occult metastatic disease

3) Therapy for known metastatic disease.

The term “ablation” specifically refers to first-line RAI treatment after surgical total thyroidectomy.

The specific target of this treatment is normal residual thyroid tissue, i.e. the remnant, (Table 1). The confusion regarding the indications of RAI ablation stems from the underestimation of the impact of the remnant volume and patterns of uptake on imaging studies following surgical total thyroidectomy. Certain surgical details need to be explained in order to clarify the remnant concept from a clinical context.

## SURGICAL ANATOMY OF TOTAL THYROIDECTOMY, SOURCES AND LOCATIONS OF THYROID REMNANTS

**Figure 1:**
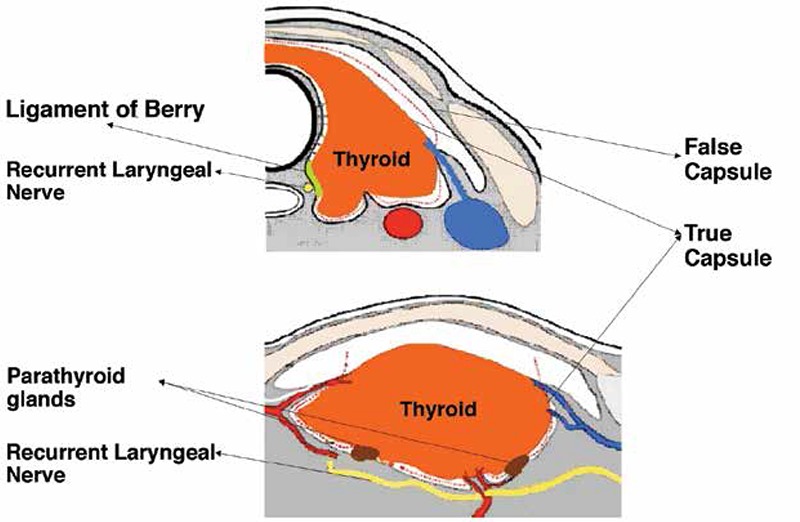
The surgical anatomy and technique of total thyroidectomy, remnant volume. This diagram adapted from Tan et al. (7) shows the fascial compartments in the thyroid space. The parathyroid glands are situated over the true capsule. The separation of the parathyroid glands off the true capsule can disrupt the vascular supply. The cranial end of the recurrent laryngeal nerve typically travels under the ligament of Berry and, at this position, is very close to the true capsule of the thyroid. A vigorous extracapsular dissection may traumatize these vital structures even if they are carefully identified and avoided during surgery

**Figure 2:**
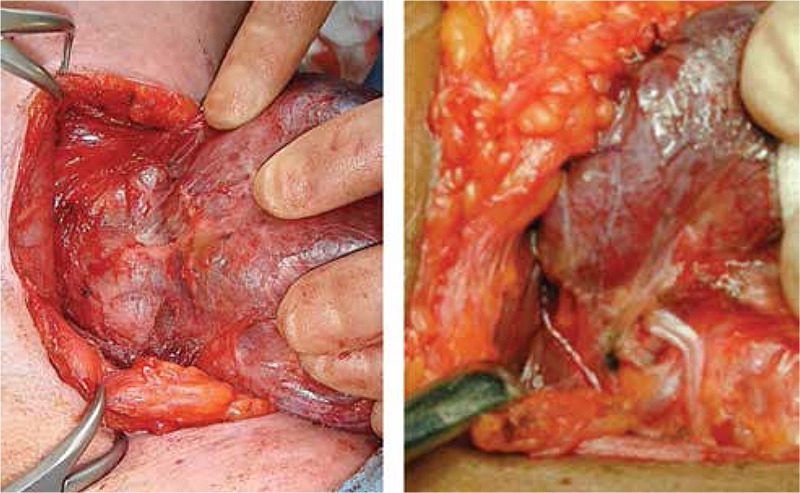
The image on the left shows a parathyroid gland lying on the thyroid capsule. The image on the right shows the recurrent laryngeal nerve under the ligament of Berry, and their very intimate relations with the true capsule of the thyroid gland

**Figure 3:**
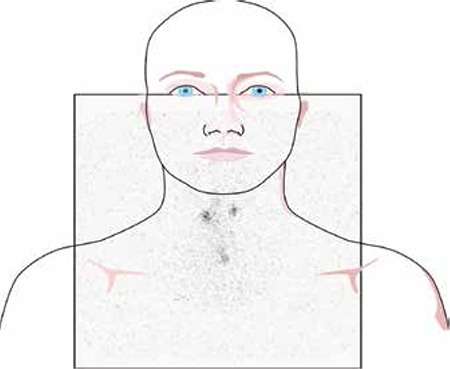
Normal postoperative radioactive iodine scan. This image shows a small focus of activity at the midline of the lower neck compatible with a midline remnant. There are also two small foci of radioactive iodine uptake in the upper neck, which are equivocal. Upon referring with the surgeon who performed the operation, these foci were confirmed to represent remnant activity beneath the superior parathyroid glands. Without knowledge of the operative findings these can easily be reported as metastatic nodal disease

The strict definition of total thyroidectomy, as it pertains to subsequent diagnostic and therapeutic considerations, is the removal of all functioning thyroid tissue. When a clinical decision is made for a lobectomy, based on a low risk disease designation, “ablation” is not part of the equation. Ablation is, in the most simplistic terms, the eradication of all functional thyroid tissue left behind after surgical total thyroidectomy. A surgical total thyroidectomy entails removing the gland in the plane outside its “true capsule” (Figure 1). The true capsule is a very fine layer. A faithful dissection over the true capsule may occasionally pose a significant challenge to the viable preservation of the parathyroid glands and safe protection of the recurrent laryngeal nerve (RLN), as these critical structures have very intimate anatomic relations with the true capsule (Figure 2). A rational definition of total thyroidectomy is, therefore, the removal of the anatomical gland with viable and non-traumatic preservation of the parathyroid glands and RLN. It is important to emphasize that injuries to these critical structures are usually caused by ischemia resulting from devascularization, or traumatic contusion due to vigorous dissection and traction, not necessarily inadvertent transection or resection. A safe dissection, when performed in a cognizant way, may result (or even require) entering inside the true capsule and leaving a focal, thin rim of functional thyroid tissue in situ. A similar challenge may be encountered dissecting the upper pole vessels. There is an intimate relationship between the external branch of the superior laryngeal nerve and the superior thyroidal artery and vein. The dissection of the thyroid upper pole can also be challenging, particularly with a high-riding position. A safe division of the upper pole vasculature could leave behind a small remnant (Figure 3). The pyramidal lobe usually has a short extension containing functional tissue that becomes a fibrotic cord as it extends cranially. It is typically divided at a reasonable length along its tract. Occasionally this pyramidal cord may also contain functioning thyroid tissue. The totality of a total thyroidectomy is reliant on the surgeon’s technique. High volume experienced surgeons can produce consistent results in terms of low remnant volume; however, the reality in community settings could vary widely. The terms near-total or sub-total thyroidectomy are disclosures of not insignificant remnant volume. Remnant thyroidectomy is accomplished by RAI ablation.

## RADIOACTIVE IODINE ABLATION: BETA-KNIFE COMPLETION THYROIDECTOMY

The therapeutic effect of I-131 works through beta particles, thus, it has been named a “beta-knife” (2). The objectives of ablation are three-fold a) Ablation eradicates all functioning thyroid tissue. With that goal accomplished, thyroglobulin (Tg) becomes a highly specific tumor marker. This simply facilitates the post-op long-term patient follow-up. b) Ablation wipes out all focal normal thyroid tissue left in-situ by the surgeon to avoid injury to laryngeal nerves and parathyroid glands. From a surgical standpoint, these small clusters of normal thyroid tissue remnants are inconsequential; however, they appear as focal areas of RAI uptake on future imaging studies and can easily be interpreted as metastatic disease. Eradication of these potential sources of misdiagnosis, while there is still open communication between the surgeon and the imager, is important. c) The post-ablation whole-body scan is considered the standard for extent of disease evaluation.

When postoperative RAI treatment is contemplated with an adjuvant intent, in addition to the remnant ablation objectives, RAI is aimed to target residual disease or occult metastatic disease. When the intent is adjuvant treatment, risk stratification becomes important in the selection of the administered activity of I-131. For remnant ablation purposes only, the risk stratification has no bearing. Complete thyroidectomy, in the strictest sense, is only possible with surgical (cold steel knife) thyroidectomy, followed by I-131 ablation (beta knife). There exists a group of patients where surgical total thyroidectomy removes all the functional remnant tissue. Although it could be argued that RAI ablation is not necessary in this group, it is challenging to identify these patients using standard clinical evaluations.

## CHALLENGES AND POTENTIAL SOLUTIONS FOR ACCURATE ASSESSMENT OF REMNANT VOLUME

The volume of the thyroid remnant is highly variable and is dependent on the surgeon’s technique; it is not typically highlighted in the operative report and is usually undetectable by ultrasound (US) or other anatomic imaging modalities such as computed tomography (CT) or magnetic resonance imaging (MRI). Serum Tg can provide some information as to the presence of functioning thyroid tissue; but the marked variability in Tg levels, which can overlap with metastatic disease, makes Tg an unreliable quantitative measure of remnant volume. Occult metastatic sites may only be detected by a post-ablation scan. Typical 1-5 mCi I-131 imaging has notoriously low sensitivity for disclosing occult foci.

**Figure 4:**
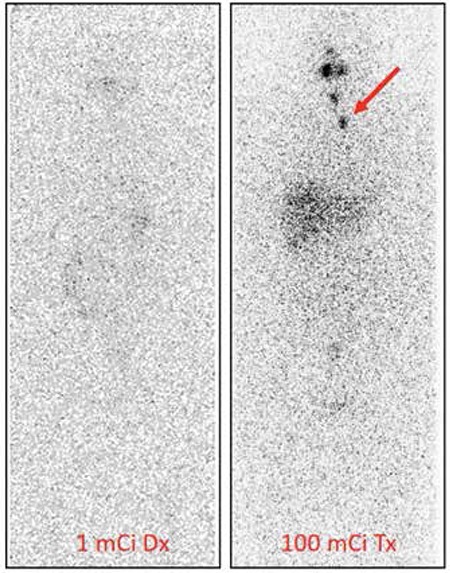
Radioactive iodine (RAI) scan with 1 mCi versus 100 mCi demonstrating occult remnant or nodal disease. The image on the left is a diagnostic whole body scan with 1 mCi of I-131 showing no abnormal RAI avid foci in the neck. The image on the right is a posttreatment scan for the same patient after therapy with 100 mCi of I-131. This image shows physiologic activity in the salivary glands as well as two RAI avid foci in the neck that were confirmed to represent nodal metastases

Postoperative/pre-ablative RAI imaging may be considered to determine the extent of residual functional thyroid tissue, or remnant volume. I-131 has become the mainstay for RAI imaging due to its availability and relatively low cost; however, one must recognize the inherent limitations of I-131 as a diagnostic imaging agent. I-131 has high energy gamma emissions with the greatest abundance of 364 keV which lead to septal penetration and limit the spatial resolution of standard gamma cameras. In addition, only low administered activities of I-131 can be given for diagnostic purposes due to concerns for stunning, especially when subsequent RAI treatment is being considered. Routine diagnostic I-131 scans are usually performed with administered activities in the range of 1-5 mCi. These low dose images are often of limited diagnostic quality and may underestimate the extent of disease evaluation. Subtle or non-visualized foci of RAI avid disease on low dose diagnostic I-131 scans may become evident on post-treatment scans with therapeutic administered activities (Figure 4). Even with higher administered activities of I-131, it can be difficult to differentiate foci of uptake in the neck as thyroid remnants versus nodal metastatic disease. RAI avid foci in the neck can be elucidated with increased confidence only after the thyroid remnant has been completely ablated. In addition, eliminating the remnant comprised of highly avid normal thyroid tissue reduces competition for RAI at metastatic sites thereby enhancing diagnostic scan sensitivity and therapeutic efficacy (3).

**Figure 5:**
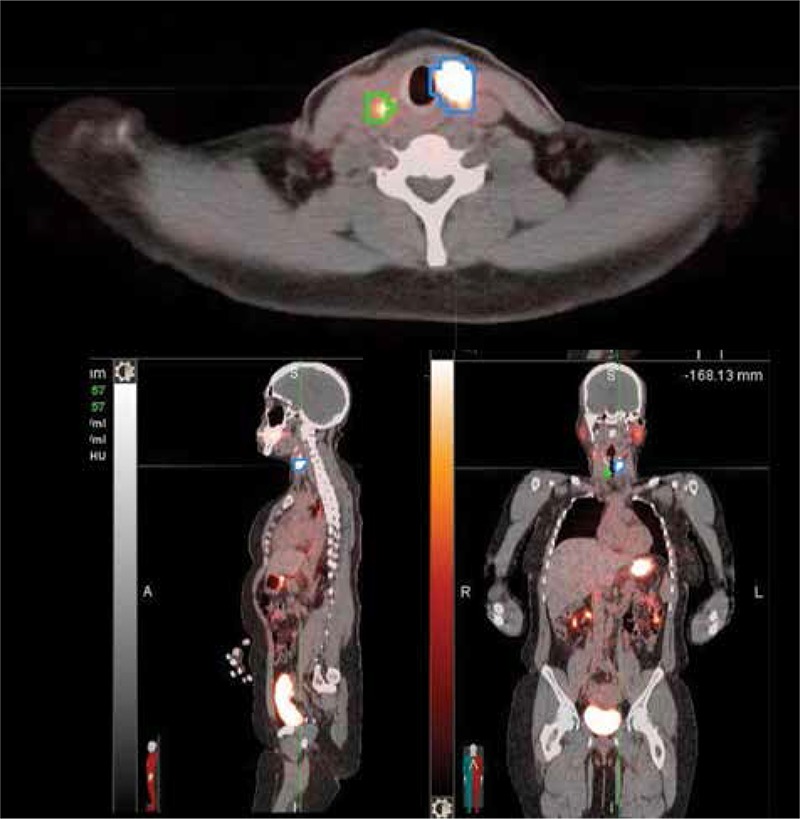
I-124 positron emission tomography/computed tomography image through the level of the thyroid bed shows regions of interest drawn around a thyroid remnant (blue) and a metastatic right level 3 lymph node (green). The remnant is not measurable by ultrasound or computed tomography. There is overestimation of functional volume (correctible) due to voxel saturation phenomenon

Although not in routine clinical use currently, I-124 imaging has been demonstrated to be a very sensitive functional imaging agent. I-124 positron emission tomography (PET)/CT has an outstanding spatial resolution in elucidating small thyroid remnants from the right or left lobes or in the distribution of the pyramidal lobe (Figure 5). Due to its anatomic precision, I-124 PET/CT can also distinguish nodal uptake from a remnant in the thyroid bed which may not be adequately visualized with other imaging modalities. I-124 has been shown to have a similar performance in detection of RAI avid lesions as compared to post-therapeutic I-131 scans. Additionally, the risk of stunning at higher administered activities of I-131 may not be observed with I-124. Therefore, I-124 may be considered the most sensitive imaging modality for assessing remnant thyroid tissue.

Perhaps the greatest merit of I-124 PET/CT is that it allows accurate quantitation of remnant volume and kinetics for the purposes of remnant dosimetry. Our group showed significant variations in cumulated activities and functional volumes of thyroid remnants between cohort patients. The maximum remnant activity ranged from 1.2 to 215.9 uCi with the total functional remnant volume (the total number of voxels within the remnant range of interest) ranging from 1 to 60 mL. The activity per volume of remnant tissue ranged from 0.036 to 11.265 uCi/mL. The total cumulated activity within the remnant ranged from 68 to 12757.3 uCi/hr (4). This variability highlights how a thyroid remnant may become a confounding factor in the postoperative management of DTC patients and why RAI ablation is key to maintaining a standard baseline for which future imaging and laboratory parameters can be compared.

## SPECIFICITY OF THYROGLOBULIN

Tg is a highly specific tumor marker when functioning thyroid tissue is no longer present, i.e. after total thyroidectomy and RAI ablation of the remnant. It has been estimated that 1 g of normal thyroid tissue releases about 1 ng/mL of Tg into the serum when thyroid-stimulating hormone (TSH) is normal and about 0.5 ng/mL when TSH is suppressed (<0.1 mIU/L) (5). The remnant volume, and thus remnant-produced Tg, is dependent on the surgical technique and potentially could be highly variable/unpredictable.

The factors influencing baseline and recombinant human TSH stimulated Tg levels in patients with metastatic DTC were investigated in a study by Robbins et al. (6). The authors found wide variations in Tg levels and considerable overlap between metastatic sites. The median baseline Tg level for cervical metastases was 2 ng/mL and increased to a median of 8 ng/mL after recombinant human TSH stimulation. The median baseline and stimulated Tg levels for mediastinal metastases were 4 ng/mL and 16 ng/mL, and for distant metastatic sites were 25 ng/mL and 180 ng/mL, respectively. The patients with thyroid remnants included in the study had a median baseline Tg level of 0.6 ng/mL, with the highest being 66 ng/mL. After recombinant human TSH, the median stimulated Tg in patients with thyroid remnants was 1.2 ng/mL but ranged as high as 250 ng/mL.

Following Tg levels in patients who have undergone total thyroidectomy but not RAI ablation is challenging. The Tg level is expected to increase by at least two-fold in the presence of a thyroid remnant under the influence of recombinant human TSH; however, the stimulated Tg level has been shown to increase almost four-fold in some patients. The inconsistent baseline Tg levels produced by the remnant along with the documented variability in stimulated Tg levels that overlap with metastatic disease make it very difficult to reliably detect occult metastasis. In addition, the natural progression of an un-ablated thyroid remnant under TSH suppression and its effect on Tg production are not clearly established. These issues can be avoided if the thyroid remnant is ablated with RAI thereby eradicating all functioning thyroid tissue and rendering Tg a very specific indicator of recurrent disease in post-surgical patients.

## THE ACTIVITY OF I-131 TO BE USED FOR REMNANT ABLATION OR ADJUVANT THERAPY

**2015 ATA Recommendation 55**

A) If RAI remnant ablation is performed after total thyroidectomy for ATA low-risk thyroid cancer or intermediate-risk disease with lower risk features (i.e., low-volume central neck nodal metastases with no other known gross residual disease or any other adverse features), a low administered activity of approximately of 30 mCi is generally favored over higher administered activities (Strong recommendation, high-quality evidence).

B) Higher administered activities may need to be considered for patients receiving less than a total or near-total thyroidectomy in which a larger remnant is suspected or in which adjuvant therapy is intended (Weak recommendation, low-quality evidence).

**2015 ATA Recommendation 56**

**Administered RAI Activity (mCi) and Radiation Absorbed Dose (Rad) Relationship**

When RAI is intended for initial adjuvant therapy to treat suspected microscopic residual disease, administered activities above those used for remnant ablation up to 150 mCi are generally recommended (in absence of known distant metastases). It is uncertain whether routine use of higher administered activities (>150 mCi) in this setting will reduce structural disease recurrence for T3 and N1 disease (Weak recommendation, low-quality evidence).

**Figure 6:**
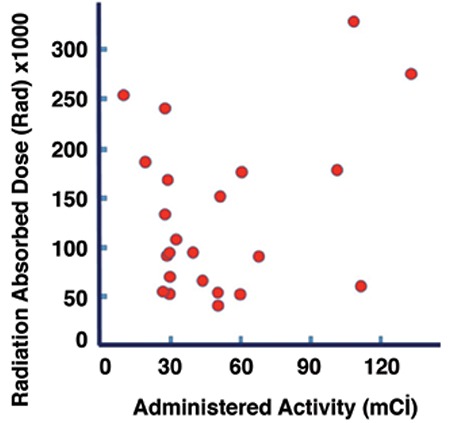
Lack of correlation between radiation dose to the remnant and millicuries of radioactive iodine administered. Adapted from (10) Hurley JR, Becker DV. The use of radioiodine in the management of thyroid cancer. Freeman LM, Weissman HS (eds). Nuclear Medicine Annual. New York, Raven Press 1983;329-384.

Currently, the most common practice in RAI ablation is to use a fixed amount of radioiodine regardless of the size of the thyroid remnant or the percentage of radioiodine uptake. Different administered activity levels have been tested over the years ranging from 30 mCi to 150 mCi. There has been a paradigm shift towards using less administered activities for RAI remnant ablation in the setting of low-risk DTC since the publication of two high-impact articles in the New England Journal of Medicine in 2012, and further propagated after release of the 2015 ATA guidelines (8,9). As paradoxical as it may seem, 30 mCi of radioiodine will completely destroy the thyroid remnant in some patients whereas 150 mCi may not be sufficient in others. The explanation is that, there is no linear relationship between the administered activity and radiation absorbed dose to the remnant. The radiation dose (in rads) depends not only on the number of millicuries administered, but also the percent uptake, the size of the remnant, and the effective half-life (T_1/2_ eff) of I-131 in the remnant. Patients with small remnants and high uptakes may receive a large radiation dose from 30 mCi whereas those with large remnants and low uptakes may receive a small and inadequate radiation dose from 150 mCi. In a 1983 dosimetric study, Becker and Hurley demonstrated a wide variation in uptake and remnant size in patients who were referred for ablation (10). There was no correlation between the number of millicuries administered and the radiation absorbed dose delivered to the remnant (Figure 6). Although this study unarguably demonstrated the unreliability of a fixed or standard amount of radioiodine for ablation, due to feasibility and logistical considerations a dosimetric approach never was adapted.

There is a clear need to develop individualized dosimetry-guided RAI ablation protocols. I-124 PET/CT offers a robust quantitative tool to achieve this goal. Active studies are being performed to determine the technical prerequisites for a time and cost-conscious standard protocol.

## SUMMARY AND RECOMMENDATIONS

Although the potential side effects of RAI are real and efforts to utilize lower administered activities for ablation are commendable, this should not overlook the opportunity for achieving complete ablation. The benefits of complete remnant ablation in the future follow-up and management of DTC patients after thyroidectomy cannot be denied. From a practical standpoint, a total thyroidectomy usually leaves behind some functioning thyroid tissue. This postsurgical thyroid remnant cannot be accurately evaluated/visualized with anatomic imaging studies like CT or ultrasound, and it can be misleading on functional RAI imaging indistinguishable from RAI avid disease in the neck. Moreover, the volume of remaining thyroid tissue is dependent upon the surgical technique and invariably and unpredictably affects the Tg level at baseline and under TSH stimulation. Avoiding RAI remnant ablation because of the risk of side effects or the reliance on underpowered, short-term prospective studies or flawed retrospective analyses fails to recognize the goals of therapy and the practical approach to the postoperative follow-up of DTC patients. The therapeutic administration of RAI to ablate the thyroid remnant allows for completion of a “true” total thyroidectomy (cold steel knife + beta knife) and improves the postoperative management of DTC patients by facilitating RAI imaging and permitting the use of serum Tg as a highly specific tumor marker.

The recommendations below incorporate genomic profiling for risk stratification and dosimetric approach for selection of administered activities for RAI ablation.

**Proposed Guidelines for RAI Ablation**

A) RAI ablation decision should be made at the time of the surgical treatment decision. Risk stratification should include genomic profiling. RAI remnant ablation is routinely recommended after total thyroidectomy, if the preoperative intent was total elimination of anatomic and functional thyroid tissue. Main consideration is the role for Tg monitoring and RAI imaging surveillance in the long-term management of patients. RAI ablation should be administered with radiation absorbed dose to the remnant tissue optimized.

B) If micropapillary cancer was incidentally reported in the surgical pathology following a total thyroidectomy, RAI ablation may be omitted if there is no histopathologic or genomic risk factor identified.

C) RAI remnant ablation is not indicated after lobectomy. If a histopathologic or genomic risk factor is identified, a completion thyroidectomy should be performed and followed by RAI ablation.

D) If the clinical intent is adjuvant treatment for suspected/known residual/metastatic disease RAI ablation should be administered in a therapeutic context, with radiation absorbed dose to the malignant disease optimized.

E) Administered activity selection should be determined based on dosimetric considerations, for both remnant ablation and adjuvant treatment indications.

## Figures and Tables

**Table 1:**
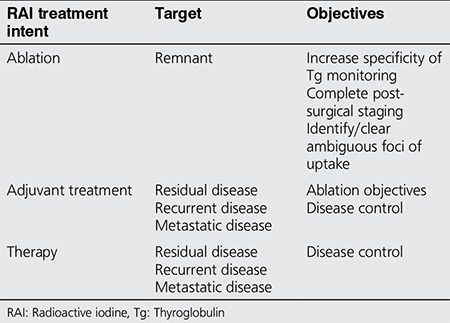
Radioactive iodine treatment objectives
